# The Emerging Role of Energy Metabolism and Neuroprotective Strategies in Parkinson's Disease

**DOI:** 10.3389/fnagi.2018.00301

**Published:** 2018-10-05

**Authors:** Janusz W. Błaszczyk

**Affiliations:** ^1^Neurophysiology, Nencki Institute of Experimental Biology, Polish Academy of Sciences, Warsaw, Poland; ^2^Human Behavior, Jerzy Kukuczka Academy of Physical Education in Katowice, Katowice, Poland

**Keywords:** Parkinson's disease, energy metabolism, brain aging, neurodegeneration, neuroprotective strategy

Over two centuries ago James Parkinson published “An Essay on the Shaking Palsy” summarizing his experience with neural pathology now known as Parkinson's disease (PD) (Parkinson, 2002). The first significant breakthrough in research on Parkinson's disease appeared 150 years later with the discovery of levodopa, a symptomatic replacement therapy for PD motor symptoms. Recently, scientific findings have forced a pivotal shift in the views on PD etiology, pointing toward an energy metabolism (Johnson and Imai, 2018; Quansah et al., 2018; Yoshino et al., 2018). The first evidence that mitochondrial dysfunctions are involved in the pathogenesis of Parkinson's disease came from parkinsonism induced by the accidental exposure of drug users to MPTP (Quansah et al., 2018; Yoshino et al., 2018). Further evidence arose from studies on post-mortem brains of PD patients that showed progressive accumulation of dysfunctional mitochondria that ultimately impaired cellular metabolism causing neuronal death. Today, there is consensus that energy metabolism plays a fundamental role as a pathomechanism of neurodegenerative diseases (Garten et al., 2015; Langston, 2017; Johnson and Imai, 2018; Quansah et al., 2018; Yoshino et al., 2018). Recent studies clearly implicate energy metabolism as a potential target for preventing and treating neurodegenerative diseases such as Parkinson's disease (Quansah et al., 2018). Evidence accumulated to-date have implicated enzymes: nicotinamide phosphoribosyltransferase (NAMPT) and nicotinamide adenine dinucleotide (NAD+) deficiency in neuronal aging and death (Garten et al., 2015; Johnson and Imai, 2018; Yoshino et al., 2018).

The perception of PD as a neurodegenerative disease initiated by energy metabolism dysfunctions has only just begun (Quansah et al., 2018). The dysfunctions appear to be genetically preprogrammed, striking initially the weakest, and most sensitive points of the nervous system. The onset of neurodegenerative disorder is manifested by several locus-specific prodromal symptoms including: depression, insomnia, loss of smell, intestinal disorders, hypertension and increased blood glucose level (Pellicano et al., 2007; Quansah et al., 2018). Unfortunately, these symptoms are usually ignored or mistreated.

The biggest obstacle impeding the development of effective therapies for PD includes a lack of understanding of its pathogenesis (Athauda and Foltynie, 2015; Yadav and Li, 2015). Even today, clinical diagnosis of PD is based on a set of motor symptoms that are linked with a decline of nigrostriatal interaction (Pellicano et al., 2007; Błaszczyk, 2017). Certainly, the main effect is the decline of the dopaminergic synaptic transmission, that in turn impairs nigrostriatal synergy with its fundamental process of the striatal interneuron turnover (Ernst et al., 2014; Błaszczyk, 2017). Apparently, the disastrous cascade of neurodegeneration can be initiated also in the GABAergic striatum (Błaszczyk, 2016). The nigrostriatal synergy adjusts the metabolism as well as the adaptive propensity of both parts of the nigrostriatal complex accordingly to their neuronal activity (Błaszczyk, 2016).

The striatum is a unique brain structure: its neurophysiological functioning depends on continuous structural remodeling that is dependent on current neurogenesis and synaptogenesis (Ernst et al., 2014; Błaszczyk, 2017). The striatal GABAergic fast spiking interneurons are characterized by a very high metabolic rate and short lifespan. Therefore, these GABAergic interneurons must be constantly replaced by neuroblasts generated in the subventricular zone niche (Ernst et al., 2014; Błaszczyk, 2017). The striatal neurogenesis and nigrostriatal synaptogenesis involve several timely coordinated metabolic processes that rely on energy supply (Ernst et al., 2014; Błaszczyk, 2017; Johnson and Imai, 2018; Quansah et al., 2018; Yoshino et al., 2018). For instance, nigrostriatal interaction is the main source of trophic signaling necessary for maintaining synaptic connections, and also produce chemo-attractants that direct the migration of neuroblasts (Ernst et al., 2014; Błaszczyk, 2017; Yoshino et al., 2018). For detailed explanation on behavioral-metabolic synergy, see Figure 1.

**Figure 1:**
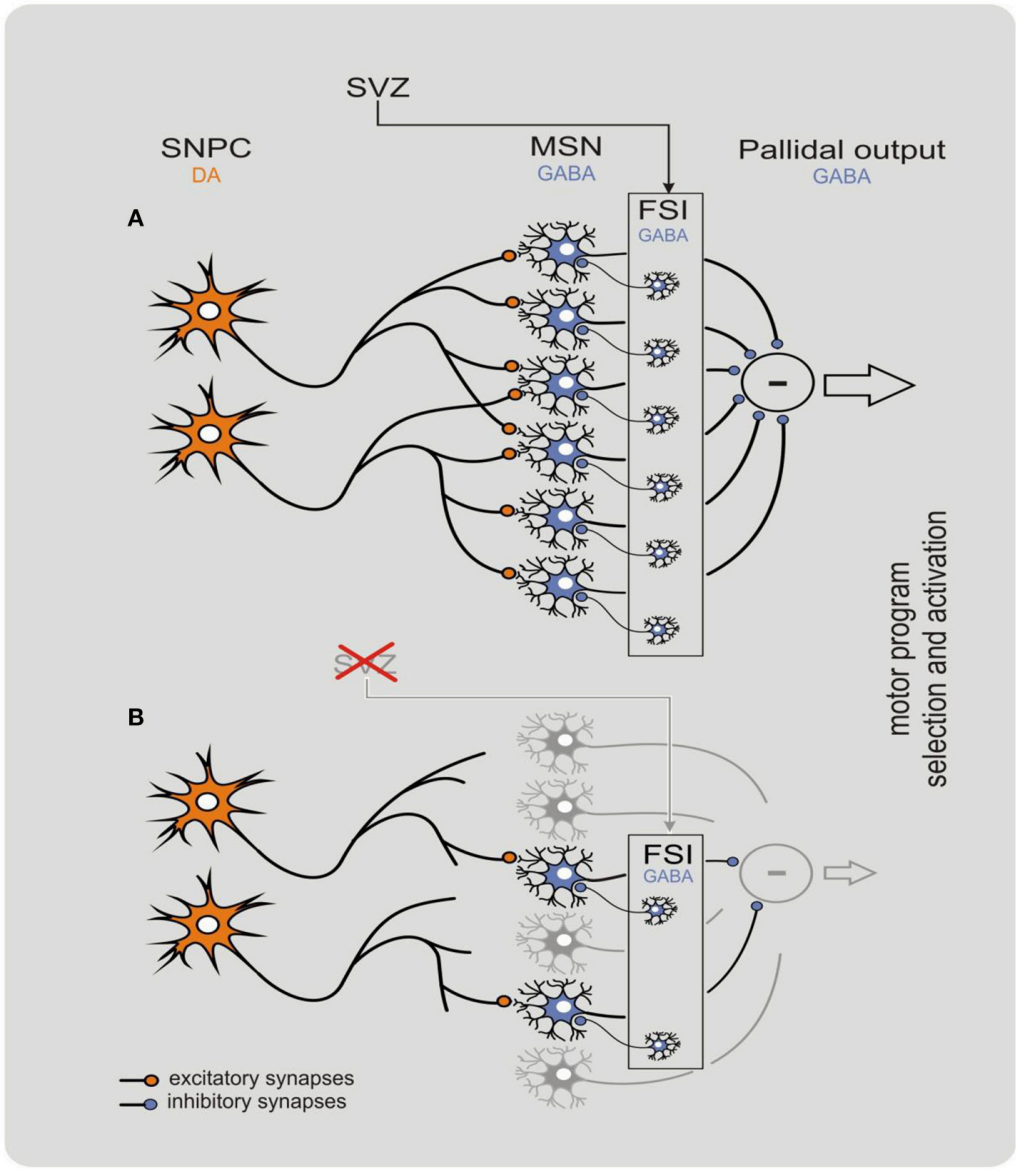
Behavioral-metabolic synergy model explaining physiology **(A)** and pathophysiology **(B)** of the nigrostriatal complex. The model clarifies how neurodegeneration of the nigrostriatal complex may be initiated in the striatum. In physiological conditions, the synergy is critically dependent on both dopaminergic input from the *substantia nigra pars compacta* (SNPC), and GABAergic network of the striatal input. The subventricular zone (SVZ) is a specialized brain area containing self-renewing population of progenitor cells that continuously replace fast spiking interneurons (FSIs) in the dorsal striatum. The process allows to maintain input threshold of the striatum limiting neuronal activity of the basal ganglia. Deficient neurogenesis within the SVZ may contribute to a decline in the nigrostriatal synergy resulting in progressive withdrawal and eventually disconnection of the dopaminergic input. Such deficiency initiates a “vicious circle” cascade of pathological events resulting in a devastating decline of nigrostriatal synergy that leads to a fatal damage to striatal input that in turn intensifies neurodegeneration of the DOPA neurons of the SNPC. In this state, the striatum loses its control over the pallidal output and several motor symptoms such as tremor, rigidity, and bradykinesia can be observed. The model reproduced with permission from Błaszczyk (2017). Copyright 2017 Acta Neurobiologia Experimentalis. Please note that within CNS the behavior-metabolic synergy has a form of repeatable sequence of intracellular biochemical processes triggered by neuronal action potentials. Such sequence must always be concluded by the process(es) of metabolic energy recovery in mitochondria. Deficit in energy metabolism may result in faulty neuronal activity increasing risk of apoptosis. In this context, impoverished SNPC activity e.g., due to natural ageing, pathology and/or reduced motor activity (hipokinesia) do potentiate neurodegeneration within the nigrostriatal system.

Collectively, recent findings may suggest the existence of a single pathomechanism of neurodegeneration i.e., the disrupted neuronal homeostasis mainly due to deficient energy metabolism (Athauda and Foltynie, 2015; Johnson and Imai, 2018; Quansah et al., 2018; Yoshino et al., 2018). The activity-dependent metabolic rate adjustment is a fundamental mechanism of cellular and tissue physiology that becomes disabled, and the vital neuronal processes and activities are progressively extinguished. Senescent neurons may remain metabolically active for a time, often continuing to perform their function in the neuronal network, albeit in a limited range. Their reduced metabolic efficiency yet still growing energy demands further disregulates energy metabolism and cellular homeostasis. Additionally, the senescent neurons excrete a plethora of molecules that affect the function of nearby cells and provoke local inflammation potentiating the destruction of the brain networks (De Virgilio et al., 2016; Quansah et al., 2018).

The mechanisms of neurodegeneration are believed to be neuron-autonomous, which implies that the same physiological events, such as mitochondrial dysfunction, dysfunction of the autophagy processes and dysregulation of calcium homeostasis, occur independently in a large number of neurons (Mosharov et al., 2009). Non-cell autonomous processes include neuroinflammation, loss of trophic support and the trans-synaptic transmission of misfolded alpha synuclein (Braak et al., 2004; Athauda and Foltynie, 2015). All of the aforementioned pathomechanisms may be initiated by a single trigger—the decline of cellular energy metabolism. One should keep in mind that several neuronal phenotypes make some brain structures more prone to neurodegeneration. In particular, neurons: (i) with long unmyelinated axons, (ii) those with a high number of synaptic connections, (iii) those with high synaptic activity, as well as (iv) the nigral dopaminergic neurons with low calbindin expression, are the most vulnerable to energy-deficiency-related metabolic crisis.

Recent searches for new and effective PD therapies focused on brain metabolism (Pellicano et al., 2007; Garten et al., 2015; Johnson and Imai, 2018; Quansah et al., 2018; Yoshino et al., 2018). The rationale for the search was strongly supported by the comprehensive concept that connects neuronal energy metabolism to the control of aging and longevity of the human brain (Quansah et al., 2018). The brain's metabolic requirements utilizes around 20% of the body's energy resources in a process that is mainly glucose dependent (Braak et al., 2004; Quansah et al., 2018). Energy from glucose oxidation is used to generate ATP, which is the main energy carrier in all living cells (Quansah et al., 2018). ATP metabolism, and thus intracellular energy metabolism, depends on nicotinamide adenine dinucleotide (NAD+). The NAD+ catalyzes redox reactions in metabolic process of glycolysis. Keeping in mind that cellular NAD+ level declines during the course of aging, maintenance of adequate NAD+ biosynthesis is paramount for neurons survival and function. Only up to 85% of NAD+ can be recycled intracellularly and the losses must be supplemented with extracellular NAD+ precursors and intermediates. Likely, the major forms of vitamin B3 constitute the most well-known NAD+ precursors that could be safely used in antineurodegeneration therapies.

Undisputedly, efficient control of brain energy metabolism is requisite for maintaining neuronal homeostasis, physiology, and survival. This neurophysiological dogma initiated intensive search for strategies targeting brain and neurons energy metabolism in attempts to find an antineurodegeneration therapy. Common for neurodegenerative disease and type 2 diabetes metabolic abnormalities including mitochondrial dysfunction and neuronal insulin resistance has directed the research toward “insulin sensitizers” e.g., MSDC-0160 (Quansah et al., 2018). Multiple studies documented that such compounds can effectively attenuate neurodegeneration by decreasing inflammatory processes (Quansah et al., 2018).

It has been discovered recently that NAD+ supplementation can effectively restore energy metabolism on both the cellular and organismal level (Wasserman, 2009; Trammell et al., 2016b; Johnson and Imai, 2018; Yoshino et al., 2018). Thus, supplementing with NAD+ intermediates and/or precursors should ameliorate the age-related functional brain deficits by counteracting neuronal aging and neurodegeneration. The newest studies have confirmed the therapeutic potential of supplementing NAD+ intermediates, such as nicotinamide riboside, providing a proof of concept for the development of new effective intervention (Athauda and Foltynie, 2015; Johnson and Imai, 2018; Yoshino et al., 2018).

In humans, NAD+ can be synthesized *de novo* from tryptophan, or from intermediates such as niacin and nicotinamide riboside (NR). NR is new form of vitamin B_3_ that functions as a precursor to NAD+ and there is growing evidence suggesting that NR may be a potent candidate to protect and improve nigrostriatal complex (Błaszczyk, 2017). NR is also a particularly attractive intermediate since it can be found in milk and dairy products (Trammell et al., 2016a,b).

We should also keep in mind that NAD+ has a critical role as the substrate of NAD-consuming enzymes including sirtuins and poly-ADP-ribose polymerases (PARPs) (Trammell et al., 2016b; Langston, 2017; Quansah et al., 2018). Whereas PARPs facilitate repair and maintenance of genomic integrity, activity of sirtuins regulates protein quality control pathways, in particular catabolism of the unfolded proteins. Unfortunately, both PARPs and the sirtuins must compete with ATP for the same, limited, and decreasing with age, pool of NAD+. Since ATP has priority in this competition, development of proteinopathy is only a matter of time. Consequently, the age-related deficit in energy metabolism well explains formation of alfa-synuclein inclusions, amyloid plaques and neurofibrillary tangles (Garten et al., 2009, 2015; Johnson and Imai, 2018). Thus intracellular accumulation of misfolded protein aggregates is caused by the age-related cellular energy crisis and the crisis is multiplied by the misfolded protein accumulation. This is typical “vicious circle.”

Given the present view of PD etiology, supplementation of key NAD+ intermediates, especially different forms of vitamin B3, can ameliorate a variety of age-associated pathophysiologies generated by metabolic energy decline (Trammell et al., 2016a,b; Johnson and Imai, 2018; Yoshino et al., 2018). Supplementation of these intermediates appears to restore NAD+ levels in both the nuclear and mitochondrial compartments of neurons (Johnson and Imai, 2018; Yoshino et al., 2018). Initial trials with oral administration of energy metabolites, however, failed to show clear and convincing benefits in PD patients. Such a result could be predicted, since energy metabolism cannot be easily recovered in senescent or already dead neurons of the nigrostriatal complex. The therapy might be only effective in the early stage of PD and should rely on long-term supplementation of NAD intermediates. There is, however, a potential “dark side” of such a therapy that should be mentioned! Due to functional-trophic coupling, the energy metabolites are rather selectively distributed in the body, giving priority to the most active tissues. Unfortunately, one of the most metabolically active is tumor tissue (Garten et al., 2009).

## Author contributions

The author confirms being the sole contributor of this work and has approved it for publication.

### Conflict of interest statement

The author declares that the research was conducted in the absence of any commercial or financial relationships that could be construed as a potential conflict of interest.

## References

[B1] Athauda D. Foltynie T. (2015). The ongoing pursuit of neuroprotective therapies in Parkinson disease. Nat. Rev. Neurol. 11, 25–40. 10.1038/nrneurol.2014.226 25447485

[B2] Błaszczyk J. W. (2016). Parkinson's disease and neurodegeneration: GABA-collapse hypothesis. Front. Neurosci. 10:269. 10.3389/fnins.2016.00269 27375426 PMC4899466

[B3] Błaszczyk J. W. (2017). Nigrostriatal interaction in Parkinson's disease: new target for treatment. Acta. Neurobiol. Exp. 77, 106–112. 10.21307/ane-2017-041 28379221

[B4] Braak H. Ghebremedhin E. Rub U. Bratzke H. Del Tredici K. (2004). Stages in the development of Parkinson's disease-related pathology. Cell Tissue Res. 318, 121–134. 10.1007/s00441-004-0956-9 15338272

[B5] De Virgilio A. Greco A. Fabbrini G. Inghilleri M. Rizzo M. I. Gallo A.. (2016). Parkinson's disease: autoimmunity and neuroinflammation. Autoimmun. Rev. 15, 1005–1011. 10.1016/j.autrev.2016.07.022 27725149

[B6] Ernst A. Alkass K. Bernard S. Salehpour M. Perl S. Tisdale J.. (2014). Neurogenesis in the striatum of the adult human brain. Cell 156, 1072–1083. 10.1016/j.cell.2014.01.044 24561062

[B7] Garten A. Petzold S. Körner A. Imai S. Kiess W. (2009). Nampt: linking NAD biology, metabolism, and cancer. Trends Endocrinol. Metab. 20, 130–138. 10.1016/j.tem.2008.10.004 19109034 PMC2738422

[B8] Garten A. Schuster S. Penke M. Gorski T. de Giorgis T. Kiess W. (2015). Physiological and pathophysiological roles of NAMPT and NAD metabolism. Nat. Rev. Endocrinol. 11, 535–546. 10.1038/nrendo.2015.117 26215259

[B9] Johnson S. Imai S. I. (2018). NAD^+^ biosynthesis, aging, and disease. F1000Research 7:132. 10.12688/f1000research.12120.1 29744033 PMC5795269

[B10] Langston J. W. (2017). The MPTP story. J Parkinsons Dis. 7(Suppl. 1), S11–S19. 10.3233/JPD-179006 28282815 PMC5345642

[B11] Mosharov E. V. Larsen K. E. Kanter E. Phillips K. A. Wilson K. Schmitz Y.. (2009). Interplay between cytosolic dopamine, calcium, and alphasynuclein causes selective death of substantia nigra neurons. Neuron 62, 218–229. 10.1016/j.neuron.2009.01.033 19409267 PMC2677560

[B12] Parkinson J. (2002). An essay on the shaking palsy. 1817. J. Neuropsychiatry Clin. Neurosci. 14, 223–36. 10.1176/jnp.14.2.223 11983801

[B13] Pellicano C. Benincasa D. Pisani V. Buttarelli F. R. Giovannelli M. Pontieri F. E. (2007). Prodromal non-motor symptoms of Parkinson's disease. Neuropsychiatr. Dis. Treat. 3, 145–52. 19300544 10.2147/nedt.2007.3.1.145 PMC2654529

[B14] Quansah E. Peelaerts W. Langston J. W. Simon D. K. Colca J. Brundin P. (2018). Targeting energy metabolism via the mitochondrial pyruvate carrier as a novel approach to attenuate neurodegeneration. Mol. Neurodegeneration 13:28. 10.1186/s13024-018-0260-x 29793507 PMC5968614

[B15] Trammell S. A. Schmidt M. S. Weidemann B. J. Redpath P. Jaksch F. Dellinger R. W.. (2016a). Nicotinamide riboside is uniquely and orally bioavailable in mice and humans. Nat. Commun. 7:12948. 10.1038/ncomms12948 27721479 PMC5062546

[B16] Trammell S. A. Yu L. Redpath P. Migaud M. E. Brenner C. (2016b). Nicotinamide riboside is a major NAD+ precursor vitamin in cow milk. J. Nutr. 146, 957–963. 10.3945/jn.116.230078 27052539 PMC6879052

[B17] Wasserman D. H. (2009). Four grams of glucose. Am. J. Physiol. Endocrinaol. Metab. 296, E11–E21. 10.1152/ajpendo.90563.2008 18840763 PMC2636990

[B18] Yadav H. P. Li Y. (2015). The development of treatment for Parkinson's disease. Adv. Parkinsons Dis. 4, 59–78. 10.4236/apd.2015.43008

[B19] Yoshino J. Baur J. A. Imai S. I. (2018). NAD^+^ intermediates: the biology and therapeutic potential of NMN and NR. Cell Metab. 27, 513–528. 10.1016/j.cmet.2017.11.002 29249689 PMC5842119

